# Catalytic Activity Is Not Required for Secreted PCSK9 to Reduce Low Density Lipoprotein Receptors in HepG2 Cells^🅂^

**DOI:** 10.1074/jbc.C700095200

**Published:** 2007-05-29

**Authors:** Markey C. McNutt, Thomas A. Lagace, Jay D. Horton

**Affiliations:** ‡ Department of Molecular Genetics, University of Texas Southwestern Medical Center, Dallas, Texas 75390; § Department of Internal Medicine, University of Texas Southwestern Medical Center, Dallas, Texas 75390

## Abstract

Proprotein convertase subtilisin/kexin type 9 (PCSK9), a member of the proteinase K subfamily of subtilases, promotes internalization and degradation of low density lipoprotein receptors (LDLRs) after binding the receptor on the surface of hepatocytes. PCSK9 has autocatalytic activity that releases the prodomain at the N terminus of the protein. The prodomain remains tightly associated with the catalytic domain as the complex transits the secretory pathway. It is not known whether enzymatic activity is required for the LDLR-reducing effects of PCSK9. Here we expressed the prodomain together with a catalytically inactive protease domain in cells and purified the protein from the medium. The ability of the catalytically inactive PCSK9 to bind and degrade LDLRs when added to culture medium of human hepatoma HepG2 cells at physiological concentrations was similar to that seen using wild-type protein. Similarly, a catalytic-dead version of a gain-of-function mutant, PCSK9(D374Y), showed no loss of activity compared with a catalytically active counterpart; both proteins displayed ~10-fold increased activity in degradation of cell surface LDLRs compared with wild-type PCSK9. We conclude that the ability of PCSK9 to degrade LDLRs is independent of catalytic activity and suggest that PCSK9 functions as a chaperone to prevent LDLR recycling and/or to target LDLRs for lysosomal degradation.

Secretory proprotein convertase (PC)^4^ enzymes are structurally related to the bacterial subtilisin-like serine protease kexin found in yeast. There are nine subtilisin-like serine proteinases in mammals designated PC1/3, PC2, furin, PC4, PC5/6, PACE4, PC7, S1P (site-1 protease), and PCSK9 (proprotein convertase subtilisin/kexin type 9) (1). PCs share a general structure that consists of a signal sequence followed by a prodomain, a conserved subtilisin-like catalytic domain, and a variable C-terminal domain (2, 3). All family members except PC2 undergo autocatalytic cleavage in the endoplasmic reticulum (ER), releasing the prodomain (4–6). PC1/3, PC2, furin, PC4, PC5/6, PACE4, and PC7 cleave at single or paired basic amino acids with the general sequence (K/R)-(*X_n_*)-(K/R)↓ . S1P and PCSK9 cleave after non-basic amino acids in the motifs R*XX*(L/K)↓ (7) and (V/I)FAQ↓ (8, 9), respectively.

The prodomain serves a dual function, assisting in both the proper folding of the protease domain and the regulation of catalytic activity (6, 10, 11). Autocatalytic cleavage of the zymogen in the ER is required for transport from this compartment (4 – 6). The excised prodomain remains noncovalently associated with the protein and inhibits aberrant protease activity (6, 11). In most PCs, the prodomain undergoes a second proteolytic processing event that relieves inhibition and unmasks enzymatic activity in the appropriate compartment (11).

Protein substrates are known for eight of the nine subtilisin-like serine proteinases. Cleavage of the substrates generally results in the production of mature bioactive proteins as well as processing intermediates or, occasionally, the inactivation of the cleaved protein (12). The only subtilisin-like serine proteinase without an identified protein substrate is PCSK9.

Like the other PCs, PCSK9 is synthesized as an inactive proenzyme and contains a triad of residues (Asp^186^, His^226^, and Ser^386^) that are required for catalytic activity (3). The ~74-kDa precursor form of PCSK9 undergoes intramolecular autocatalytic cleavage in the ER, which produces a 14-kDa prodomain and a ~60-kDa catalytic fragment (8, 9). The cleaved prodomain remains associated with the catalytic domain forming a complex that is transported to the Golgi apparatus and subsequently secreted (3, 9). Several studies have shown that the secreted mature form of PCSK9 contains an intact prodomain, with no evidence of secondary proteolytic processing (9, 13, 14).

Much attention has been focused on the biological role and potential substrates of PCSK9 since the discovery that gain-of-function mutations in *PCSK9* cause an autosomal dominant form of familial hypercholesterolemia (15). Studies in mice in which PCSK9 was overexpressed demonstrated that PCSK9 mediates the destruction of LDL receptor (LDLR) protein in liver, the primary receptor responsible for LDL cholesterol clearance from plasma (9, 16–18). Conversely, humans with loss-of-function mutations in *PCSK9* have lower plasma LDL cholesterol levels, and PCSK9 knock-out mice have increased LDLR protein expression in liver (19, 20).

The genetic data from humans and the *in vivo* studies in mice demonstrate that PCSK9 reduces the number of the LDLRs; however, the mechanism by which PCSK9 carries out this function is only partially known. Evidence is consistent with the secreted form of PCSK9 binding directly to the LDLR and resulting in degradation of the receptor (13, 21). Zhang *et al.* (22) localized the binding site of PCSK9 in the LDLR to the first epidermal growth factor-like repeat (EGF-A) of the extracellular domain and showed that PCSK9 binding to this site is required for LDLR degradation. For the secreted form of PCSK9 to destroy LDLRs, the PCSK9-LDLR complex must be internalized into endosomal/lysosomal compartments (13).

Although binding and internalization of PCSK9 and the LDLR are required for PCSK9 to promote degradation of the receptor, it is not known whether PCSK9 acts catalytically in the process to cleave either the LDLR or an accessory protein that affects LDLR stability. Inactivation of the catalytic activity using routine mutagenesis results in either PCSK9 remaining sequestered in the ER or in improper folding of the protein. Therefore, we have addressed this question by expressing the prodomain and an inactive catalytic domain in *trans* in cells and purifying the resultant recombinant protein complex from the medium. Using this reagent we provide evidence that PCSK9 catalytic activity is not required for PCSK9 to bind and degrade LDLRs in cultured human hepatoma HepG2 cells.

## EXPERIMENTAL PROCEDURES

### Construction of Trans and Mutant PCSK9 Expression Vectors—

Details of plasmid construction are in the supplemental material.

### Transient and Stable Transfection of Human Embryonic Kidney (HEK) 293 Cells with PCSK9 Mutants—

HEK 293 cells were transfected using Lipofectamine 2000 transfection reagent (Invitrogen). For stable lines expressing *trans*-PCSK9, HEK 293S cells (23) were stably transfected with two plasmids, one encoding the prodomain and the other encoding a catalytic fragment. Additional details of the transfections are in the supplemental material.

### Antibodies and Immunoblot Analysis—

Monoclonal antibody 3C12 (IgG subclass 1) resulted from immunization with full-length human PCSK9 (24). Recombinant human PCSK9 prodomain purified from *Escherichia coli* was used to produce monoclonal antibody 1A1 (IgG subclass 2a). Both antibodies recognize epitopes in the prodomain of PCSK9. Additional antibodies used are in the supplemental material.

### PCSK9 Activity Assays—

HepG2 cells were incubated with PCSK9 and proteins prepared as described (13). Ligand blotting of purified human LDLR was performed as described (13) using the buffer described in the supplemental material.

## RESULTS

**FIGURE 1.**
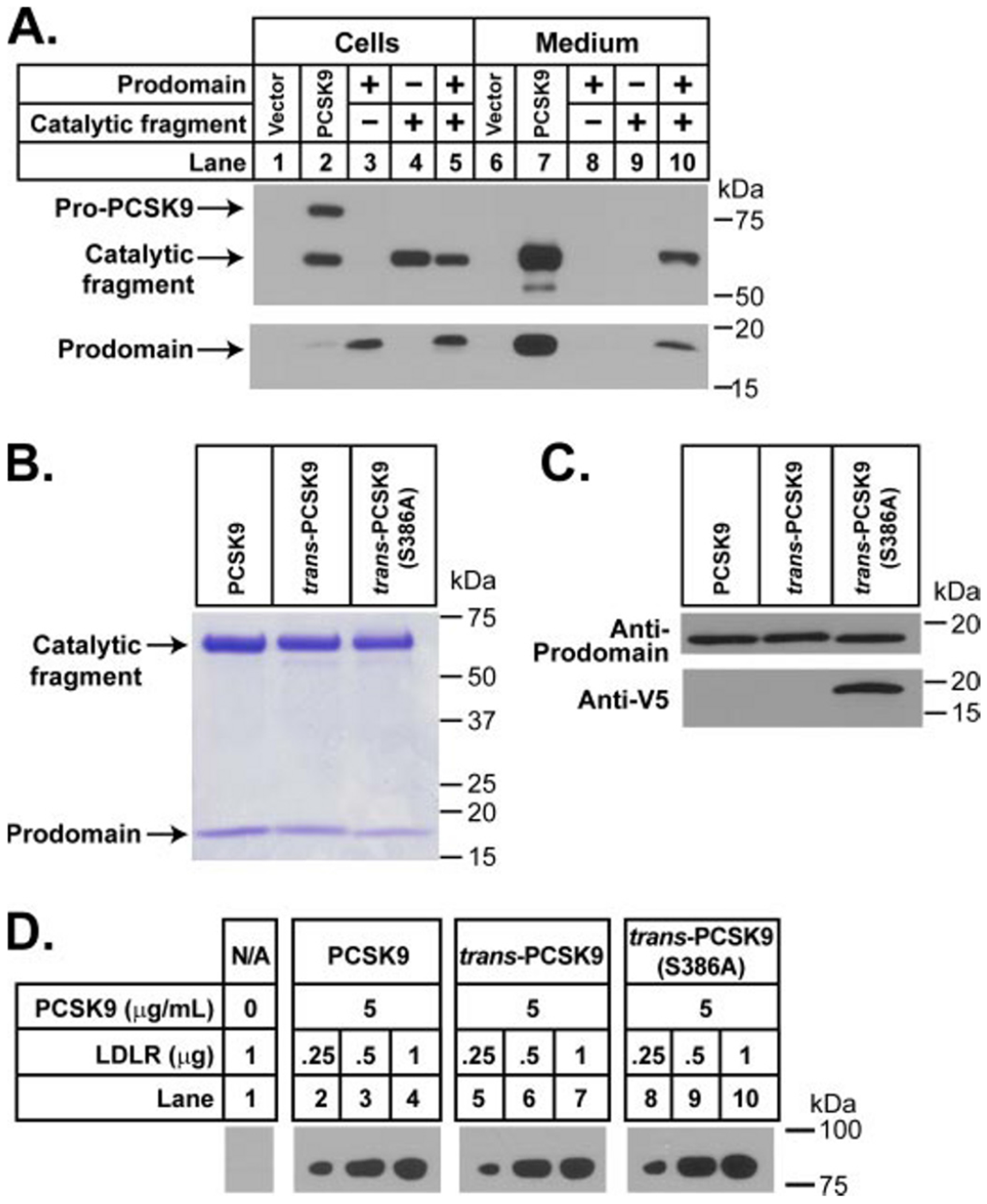
PCSK9 is secreted and binds the LDLR when expressed in *trans*. *A*, HEK 293 cells were transiently transfected with wild-type PCSK9, the prodomain of PCSK9, catalytic fragment of PCSK9, or both the prodomain and catalytic fragment of PCSK9. Medium and cell extracts were subjected to SDS-PAGE and immunoblot analysis. The catalytic fragment of PCSK9 was detected with IgG-15A6 and the prodomain with IgG-1A1. *B*, PCSK9, *trans*-PCSK9, and *trans*-PCSK9(S386A) were purified from stably transfected HEK 293S cells. 5 *μ*g of each protein was resolved on 4–15% SDS-PAGE and stained with Coomassie Brilliant Blue R-250. *C*, purified wild-type PCSK9, *trans*-PCSK9, and *trans*-PCSK9(S386A) were analyzed for the presence of the prodomain by 4 –15% SDS-PAGE and immunoblotting. The prodomain was detected with the IgG-3C12 anti-prodomain and anti-V5 antibodies. *D*, the indicated amount of purified extracellular domain of the LDLR was resolved under nonreducing conditions by SDS-PAGE and transferred to nitrocellulose. Filters were incubated with the indicated purified PCSK9 (5 *μ*g/ml) or buffer control (*N/A*) for 1 h and then incubated with 2.5 *μ*g/ml anti-FLAG M2 antibody.

Among the PC family of mammalian serine proteases, auto-catalytic cleavage of the prodomain is required for entry into the secretory pathway. Accordingly, mutating the conserved serine (Ser^386^) of the catalytic triad in PCSK9 prevents autocatalytic cleavage resulting in retention of the protein within the ER (8, 17). To determine whether catalytic activity is required for exogenous PCSK9 to destroy LDLRs, an alternative strategy was employed to obtain secreted catalytically inactive PCSK9. An expression plasmid was constructed that contained the signal peptide and prodomain of PCSK9 followed by a V5 epitope tag. A second expression plasmid was constructed that contained the signal sequence, catalytic, and C-terminal portions of PCSK9 followed by a FLAG tag. The two plasmids were co-transfected into HEK 293 cells to determine whether the PCSK9 fragments expressed in *trans* could associate and be secreted from the cell. As shown in Fig. 1A, the prodomain and catalytic fragments were similarly expressed in the transfected cells (*lanes 3–5*); however, secretion of either peptide only occurred when both fragments were co-expressed (*lane 10*). The ability to purify PCSK9 by co-expressing the prodomain and catalytic fragment of PCSK9 now provided a means to mutate key amino acid residues of the catalytic triad to determine whether catalytic activity was required for PCSK9 activity.

An HEK 293S cell line was subsequently established that stably expressed the “*trans*-PCSK9” or a *trans*-PCSK9 protein containing an alanine substituted for the serine at position 386 (S386A) of the catalytic triad. This amino acid change abolishes the autocatalytic cleavage of PCSK9 (3, 17). From these cell lines, large amounts of the recombinant purified protein could be obtained to characterize their binding properties and test for activity. Secreted *trans*-PCSK9 proteins were purified using the FLAG epitope as previously described (13). Fig. 1B shows that the catalytic fragment and prodomain co-purify with both proteins expressed in *trans*, indicating the two fragments interact and are secreted together in a manner similar to that of the wild-type PCSK9 purified from HEK 293S cells stably transfected with a single plasmid encoding the full-length PCSK9 protein. The alanine to serine change at position 386 of the purified protein was confirmed by sequencing using mass spectrometry (data not shown). Further evidence that the S386A mutation produces a catalytically inactive protein was gained from the immunoblot data using an antibody that recognizes the V5 tag present at the C terminus of the prodomain. Immunoblot of purified PCSK9 or *trans-*PCSK9 using an anti-V5 or anti-PCSK9 prodomain antibody revealed that only the *trans-* PCSK9(S386A) retained the V5 tag (Fig. 1C). This indicates that the wild-type *trans*-PCSK9 could cleave the V5 tag from the prodomain and the *trans*-PCSK9(S386A) could not. The sequence of the cleavage site in *trans*-PCSK9 (VFAQ^]152^↓GKP) was verified by mass spectrometry and was found to occur at the same residue at which the prodomain is normally cleaved from full-length PCSK9 (VFAQ^152^↓SIP).

We first determined whether the secreted recombinant *trans*-PCSK9 proteins could bind to the LDLR by ligand blotting using purified LDLR protein. Fig. 1D shows that the purified *trans*-XSXSPCSK9 and *trans*-PCSK9(S386A) proteins bound the LDLR with affinities similar to that measured with purified PCSK9 protein derived from a single plasmid encoding the full-length protein. We next tested whether catalytic activity was required for PCSK9 to degrade LDLRs when added to the medium of HepG2 cells cultured in sterol-depleted medium to induce LDLR expression (13). After incubation with purified PCSK9, the surface proteins of the cells were covalently modified with a cell-impermeable biotinylation reagent and then isolated using streptavidin beads. Total cellular LDLRs in whole-cell extracts and cell surface LDLRs were measured by SDS-PAGE and immunoblotting (13). The amounts of whole-cell and cell surface PCSK9 were also measured by SDS-PAGE and immunoblotting using an anti-FLAG antibody that detected only exogenously added PCSK9.

**FIGURE 2.**
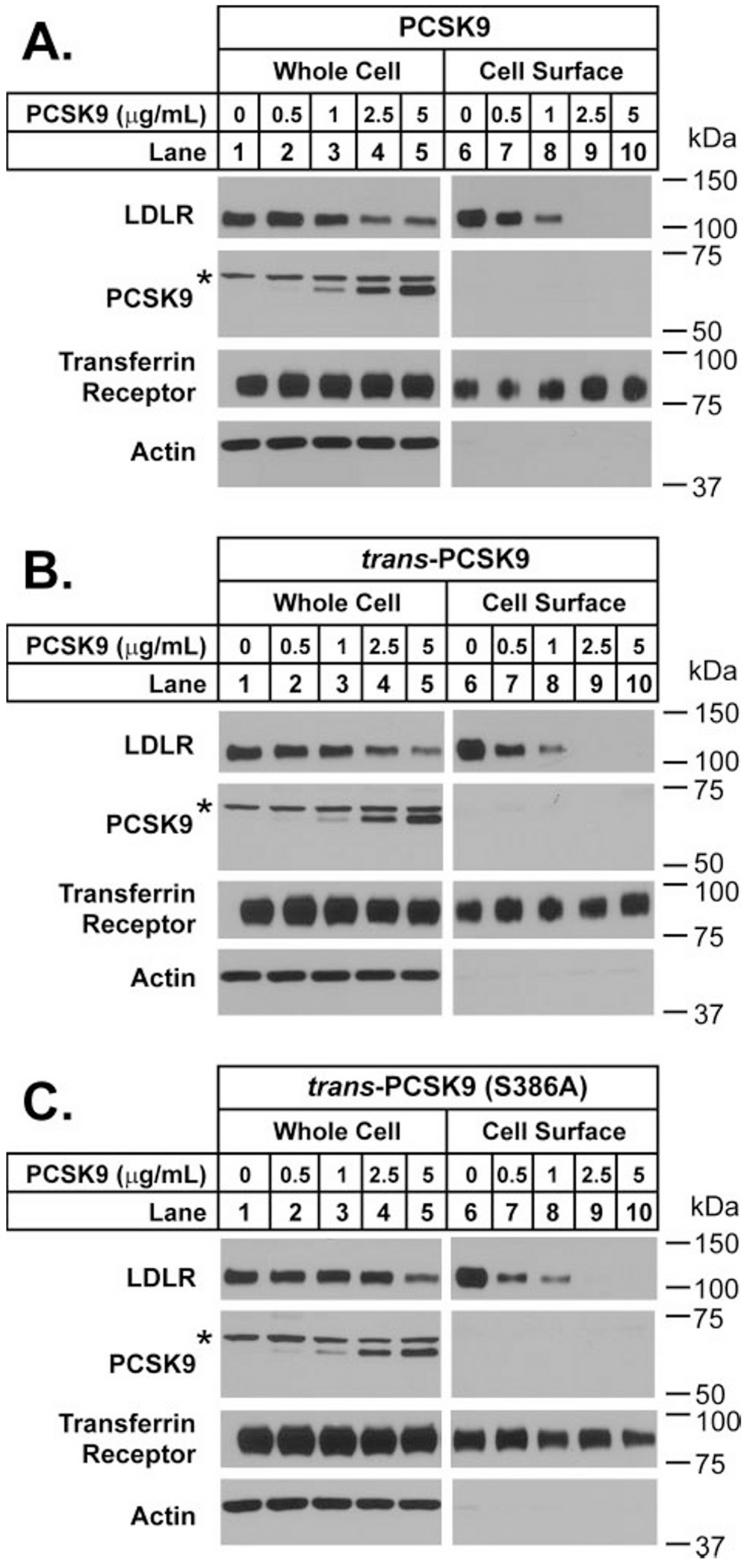
Catalytically inactive PCSK9 degrades the LDLR when added to HepG2 cells. Cells were cultured with the indicated concentrations of PCSK9 (*A*) *trans*-PCSK9 (*B*), or *trans*-PCSK9(S386A) (*C*) for 4 h. Cell surface proteins were biotinylated and whole-cell and cell surface extracts resolved by 8% SDS-PAGE. Immunoblot analysis was performed for LDLR using IgG-HL1 and for FLAG-tagged PCSK9 using anti-FLAG M2. Transferrin receptor was used as control for loading and nonspecific protein degradation. Actin was detected as a control for loading and biotinylation of non-cell surface proteins. Relative levels of whole-cell LDLR protein were: *A*, 1.0, 1.0, 0.7, 0.5, and 0.5 in *lanes 1–5*, respectively; *B*, 1.0, 0.8, 0.8, 0.7, and 0.5 in *lanes 1–5*, respectively; *C*, 1.0, 0.9, 0.9, 0.9, and 0.6 in *lanes 1–5*, respectively. Relative levels of cell surface LDLR protein were: *A*, 1.0, 0.9, 0.4, N.D., and N.D. in *lanes 6–10*, respectively; *B*, 1.0, 0.5, 0.2, N.D., and N.D. in *lanes 6–10*, respectively; *C*, 1.0, 0.4, 0.2, N.D., and N.D. in *lanes 6–10*, respectively. *, indicate cross-reacting proteins.

Fig. 2 shows that PCSK9 purified from the full-length plasmid (PCSK9), *trans*-PCSK9, and *trans*-PCSK9(S386A) had nearly identical potencies in reducing the number of LDLRs on the cell surface at all concentrations tested without affecting another cell surface receptor, the transferrin receptor (compare *lanes 6–10* in Fig. 2, A–C). FLAG-tagged PCSK9 was detected in whole-cell extracts in a concentration-dependent manner (Fig. 2, A–C, *lanes 2–5*) but was not detected among the biotin-labeled cell surface proteins (*lanes 7–10*), suggesting that most of the cell-associated PCSK9 had been internalized. Of note, catalytically inactive PCSK9 functioned to degrade LDLRs at 0.5 *μ*g/ml, a concentration similar to that found in human plasma (Fig. 2C, *lane 7*) (13).

These results indicate that catalytic activity of PCSK9 is not required to degrade LDLRs when PCSK9 is added exogenously to HepG2 cells. Previously, point mutations in human PCSK9 were identified that resulted in a gain-of-function and hypercholesterolemia (15). One gain-of-function PCSK9 mutation, PCSK9(D374Y), binds to the LDLR with ~25-fold greater affinity and is ~10-fold more active in reducing LDLRs than the wild-type protein (13, 14). If the hypothesis that binding of PCSK9 to LDLR, but not catalytic activity, is required for PCSK9 to degrade the LDLR is correct, then the introduction of the D374Y mutation into the catalytically inactive PCSK9(S386A) should still confer increased binding and enhanced degradation capacity. To test this possibility, an HEK 293S cell line was established that stably expressed PCSK9 in *trans* and that contained both the D374Y and S386A mutations in the catalytic fragment (*trans*-PCSK9(D374Y-S386A)).

**FIGURE 3.**
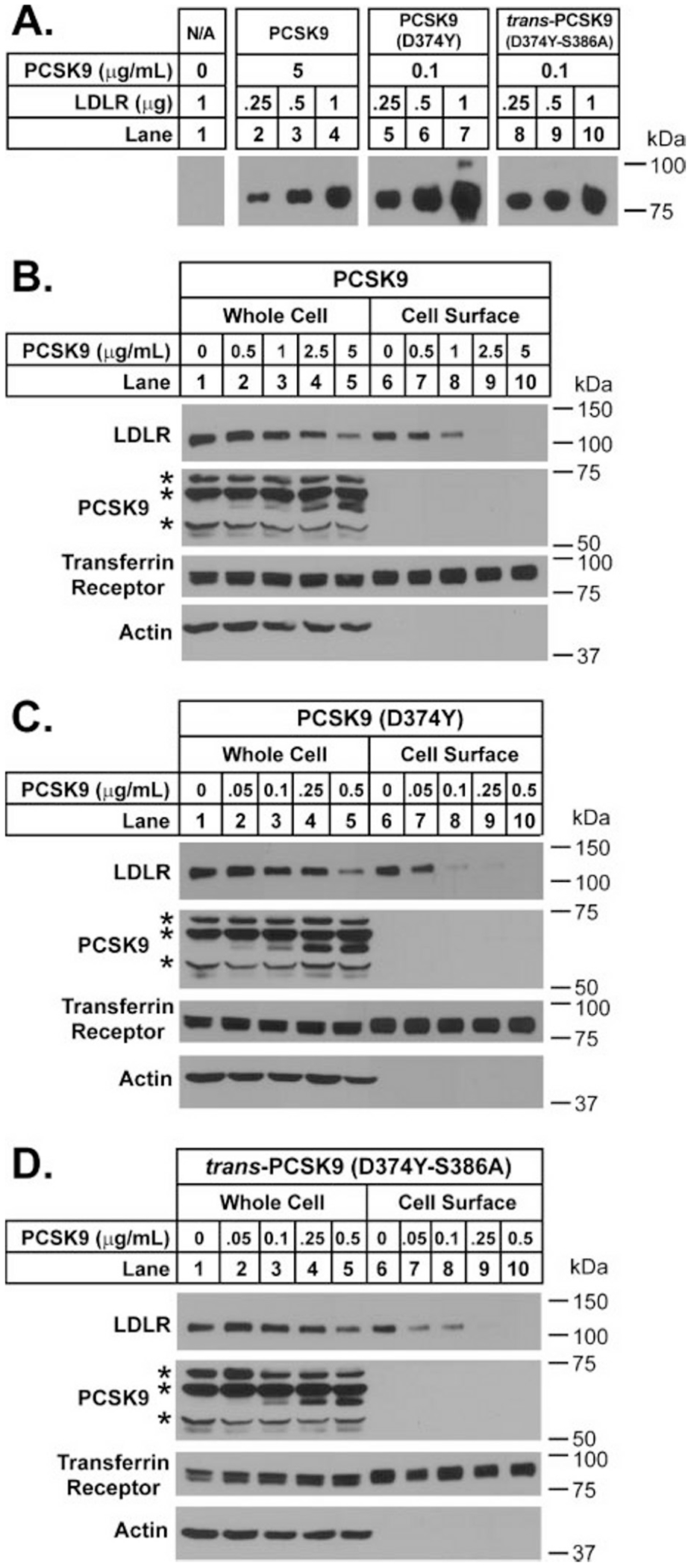
The D374Y gain-of-function mutation increases the ability of catalytically inactive *trans*-PCSK9 to bind and degrade LDLRs. *A*, ligand blotting of the extracellular domain of LDLR with 5 *μ*g/ml PCSK9 or 0.1 *μ*g/ml PCSK9(D374Y) or *trans*-PCSK9(D374Y-S386A) was performed as described in the legend for Fig. 1D. HepG2 cells were treated with the indicated concentration of wild-type PCSK9 (*B*), PCSK9(D374Y) (*C*), or *trans*-PCSK9(D374Y-S386A) (*D*) for 4 h, and LDLR, PCSK9, transferrin receptor, and actin protein levels were measured by immunoblot analysis as described in the legend for Fig. 2. Relative levels of whole-cell LDLR protein were: *B*, 1.0, 0.9, 0.7, 0.6, and 0.3 in *lanes 1–5*, respectively; *C*, 1.0, 0.9, 0.8, 0.7, and 0.4 in *lanes 1–5*, respectively; *D*, 1.0, 1.2, 0.9, 0.7, and 0.4 in *lanes 1–5*, respectively. Relative levels of cell surface LDLR protein were: *B*, 1.0, 0.9, 0.5, 0.1, and N.D. in *lanes 6–10*, respectively; *C*, 1.0, 0.8, 0.4, 0.3, and N.D. in *lanes 6–10*, respectively; *D*, 1.0, 0.4, 0.4, 0.1, and N.D. in *lanes 6–10*, respectively. *, indicate cross-reacting proteins.

The binding of the *trans*-PCSK9(D374Y-S386A) was first evaluated by ligand blotting (Fig. 3A). The affinity of *trans*-PCSK9(D374Y-S386A) for the LDLR was similar to that of the D374Y protein, both of which had an apparent higher affinity for LDLR than the wild-type PCSK9 protein. When added to the medium of HepG2 cells at one-tenth the concentration of wild-type PCSK9, the *trans*-PCSK9(D374Y-S386A) and the PCSK9(D374Y) proteins were able to degrade the LDLRs on the cell surface (Fig. 3, C and D, *lanes 7–10*). These data further support the conclusion that the binding of PCSK9 to the LDLR facilitates the degradation of the LDLR through a mechanism that does not require proteolytic activity of PCSK9.

## DISCUSSION

In this report, we demonstrate that introducing a mutation in PCSK9 that abolishes catalytic activity but does not interfere with the secretion of the protein failed to alter the mutant protein’s ability to bind to the LDLR or to mediate the destruction of LDLRs when added to the medium of cultured HepG2 cells. These results support a model in which exogenous PCSK9 binds to the LDLR, which then either targets the LDLR to the lysosome for degradation or prevents the recycling of the receptor in a manner that is independent of inherent catalytic activity of the protein. These data indicate that unlike other PCs, PCSK9 is unique as a subtilisin-like serine protease in that the protein carries out a biological function that is independent of its proteolytic activity.

Studies have demonstrated that PCSK9 and the LDLR interact directly and that the association of PCSK9 with the cell surface and its subsequent internalization is dependent upon the presence of LDLRs (13, 22). In addition, both proteins co-localize to a late endocytic/lysosomal compartment, and internalization is required for PCSK9 to reduce LDLR protein levels because this activity is blocked in the absence of autosomal recessive hypercholesterolemia (ARH), an endocytic adaptor protein required in hepatocytes for LDLR internalization in clathrin-coated pits (13, 22). Inasmuch as PCSK9 is a member of the proteinase K subfamily of subtilisin-related serine endoproteases, it seemed likely that PCSK9 cleaved the LDLR, which then facilitated degradation. A second possibility was that PCSK9 cleaved another as yet unidentified protein that ultimately mediated the destruction of LDLRs. The data of Fig. 2 indicate that catalytic activity is not required for PCSK9 to mediate the destruction of LDLRs when added exogenously to HepG2 cells. The data of Fig. 3 further support the conclusion that binding of PCSK9 to the LDLR is sufficient to target LDLRs for degradation, because inactivating the catalytic site in the hyperactive protein PCSK9(D374Y) did not change the ~10-fold higher specific activity of the protein (13). These data also support the conclusion that the gain-of-function of PCSK9(D374Y) is a result of its increased affinity for the LDLR.

Previous studies have suggested that cleavage of PCSK9 is required for the protein to be secreted from cells (9). We have found that if catalytically inactive mutants are highly overexpressed, a significant amount of unprocessed PCSK9 is secreted into the medium. The secreted full-length but catalytically inactive protein was purified and tested *in vitro* as described in Figs. 1 and 2; however, the uncleaved PCSK9 protein did not bind to the LDLR nor did it reduce LDLRs in HepG2 cells (data not shown). Thus, cleavage of the prodomain may be required for PCSK9 to adopt a conformation that mediates LDLR binding.

The data of Fig. 1 demonstrates that the PCSK9 prodomain is capable of performing a chaperone function in *trans*, as evidenced by the ER exit and secretion of *trans*-PCSK9 and the uncompromised ability of the protein to bind to the LDLR. Previously, it has been shown that mature, active furin can be formed in monkey kidney BSC-40 cells by the co-expression of the furin prodomain and a furin fragment lacking the prodomain on separate plasmids (11). In the case of PCSK9, the ability of the prodomain to function in *trans* allowed for studies of the effect of catalytic function on LDLR degradation independent of effects on PCSK9 trafficking in the secretory pathway. The crystal structure of PCSK9 was recently solved at 2.0-Å resolution (14). The prodomain failed to contain an apparent target loop that is found in the prodomains of other proprotein convertases and typically is the site of a second cleavage event that results in exposure and activation of the protease. The absence of a target loop in the prodomain of PCSK9 has been interpreted as evidence that catalytic activity may not be involved in the LDLR-lowering function of PCSK9 (14). The current studies provide direct experimental evidence that exogenously added PCSK9 reduces LDLRs in a manner that requires LDLR binding but not catalytic activity.

Studies by Maxwell *et al*. (25) showed that overexpression of PCSK9 in HepG2 cells induces degradation of LDLRs intracellularly in a post-ER compartment, and Nassoury *et al.* (26) have suggested that the two proteins interact in the ER and co-localize in the Golgi apparatus in hepatocytes. The results of our studies do not preclude the possibility that PCSK9 can function intracellularly in a manner that requires catalytic activity.

Previously, Cohen *et al.* (19) reported that one of every 50 African-Americans inherits a nonsense mutation in *PCSK9* that lowers LDL cholesterol levels by ~40%. Ina 15-year prospective study, non-sense mutations in PCSK9 reduced LDL cholesterol levels by 28%, which was associated with a reduction in the frequency of coronary heart disease of 88% in African-Americans (27). In addition, individuals that carry these cholesterol-lowering loss-of-function mutations in PCSK9 appear to have a normal life span (27). Thus, genetic data from humans with loss-of-function mutations in *PCSK9* validate PCSK9 as a potential target for the treatment of hypercholesterolemia and suggest that inhibitors of the protease would be of therapeutic benefit. The lack of a requirement for PCSK9 catalytic activity in reducing LDLRs has important implications for the development of PCSK9 inhibitors. It has been shown previously that LDLR levels do not change when a catalytically dead enzyme that is not secreted is expressed in liver (17) or in cultured liver cells (28). Thus, the current data now suggest that inhibitors of PCSK9 catalytic activity will have to function intracellularly in the ER to block PCSK9 secretion and, hence, its ability to reduce LDLR protein levels.

## Supplementary Material

supp

## Footnotes

4 The abbreviations used are: PC, proprotein convertase; ER, endoplasmic reticulum; LDL, low density lipoprotein; LDLR, LDL receptor; HEK, human embryonic kidney; N.D., not detected.
