# Supplementary material for: Catalytic Activity Is Not Required for Secreted PCSK9 to Reduce Low Density Lipoprotein Receptors in HepG2 Cells
Source: J Biol Chem. Author manuscript; Available in PMC 2026 Jun 18. (PMC13276728; doi:10.1074/jbc.C700095200)
Supplement: supp [file NIHMS2180735-supplement-supp.pdf]

Supplemental EXPERIMENTAL PROCEDURES

Construction of Trans and Mutant PCSK9 Expression Vectors— All DNA manipulations were performed using standard molecular biology techniques (1). pCMV-PCSK9(AA1-152)-V5, a vector encoding the signal sequence and prodomain of PCSK9 (amino acids 1-152) and a C-terminal V5 epitope tag (GKPIPNPLLGLDST), was generated by PCR using the pCMV-PCSK9-FLAG vector (2) as a template and the following primers: forward primer 5'-GGAATTCGCCACCATGGGCACCG-3'; and reverse primer 5'-CTTATCACGTAGAATCGAGACCGAGGAGAGGGTTAGGGATAGGCTTACCCTGGGCA AAGACAGAGGAGTCCTC-3'. The PCR fragment was cloned into pCR2.1-TOPO (Invitrogen) and the insert was then digested with *Hind*III and *Xba*I before ligation into pcDNA3.1/Hygro(+) (Invitrogen). A vector containing a deletion of the prodomain (amino acids 31-152) of PCSK9 (pCMV-PCSK9Δ31-152-FLAG) was generated through site-directed mutagenesis of the pCMV-PCSK9-FLAG vector using the oligonucleotide 5'-CCCGCGGGCGCCCGTGCGAGCATCCCGTGGAACCTG-3'. A S386A change was introduced into the pCMV-PCSK9Δ31-152-FLAG vector by site-directed mutagenesis using the primer 5'-ACAGAGTGGGACAGCCCAGGCTGCTGCCC-3'; the resulting plasmid was designated pCMV-PCSK9Δ31-152(S386A)-FLAG. Site-directed mutagenesis was then used to introduce a D374Y amino acid in pCMV-PCSK9Δ31-152(S386A)-FLAG to produce the plasmid (pCMV-PCSK9Δ31-152(D374Y-S386A)-FLAG) as described (3).

Tissue Culture Medium—Medium A contained Dulbecco's Modified Eagle Medium (DMEM) (Cellgro) supplemented with 100 units/ml penicillin, 100 μg/ml streptomycin sulfate and 1 g/L glucose. Medium B contained Medium A supplemented with 10% (v/v) fetal calf serum (FCS). Medium C contained Medium A supplemented with 1% Insulin-Transferrin-Selenium (ITS) (Cellgro). Medium D contained DMEM supplemented with 2.5% (v/v) newborn calf lipoprotein-deficient serum (NCLPDS), 100 units/ml penicillin, 100 μg/ml streptomycin sulfate, 10 μM sodium compactin, 50 μM sodium mevalonate, and 4.5 g/L glucose.

Transient Transfection of Human Embryonic Kidney (HEK) 293 Cells with PCSK9 Mutants—HEK 293 cells were plated at 5 x 10^5 cells/60-mm dish in Medium B on day 0. On day 3, plasmids were transfected using Lipofectamine 2000 transfection reagent (Invitrogen) per the manufacturer's instructions. For transfection of the full-length PCSK9 plasmid, 0.4 μg of DNA was co-transfected with 1.2 μg of empty vector DNA. For *trans*-PCSK9 experiments, 0.4 μg of pCMV-PCSK9Δ31-152-FLAG was co-transfected with 1.2 μg of pCMV-PCSK9 (AA1-152)-V5. On day 4, Medium B was replaced with Medium C. On day 5, cells and medium were harvested. Cells were washed 3 times in cold phosphate-buffered saline and lysed in buffer A (50 mM Tris-HCl, pH 7.4, 150 mM NaCl, 5 mM EDTA, 5 mM EGTA, 1% (v/v) NP-40, and 0.5% (w/v) sodium deoxycholate) with protease inhibitors (1 mM dithiothreitol, 1 mM PMSF, 0.5 mM Pefabloc, 10 μg/ml leupeptin, 5 μg/ml pepstatin A, 25 μg/ml ALLN, and 10 μg/ml aprotinin). Samples were subjected to SDS-PAGE and immunoblot analysis as described (2).

Stable Transfection and Purification of Epitope-tagged PCSK9 Mutants—For stable lines expressing *trans*-PCSK9, HEK 293S cells were cultured in Medium B in 100-mm dishes and transfected with 0.5 μg of pCMV-PCSK9(AA1-152)-V5 using Lipofectamine 2000 transfection reagent (Invitrogen). Colonies surviving hygromycin selection were isolated and expression of PCSK9 prodomain was verified by immunoblot analysis using an anti-V5 antibody (see below). The colony with highest prodomain expression was transfected with 0.5 μg of pCMV-PCSK9Δ31-152-FLAG, pCMV-PCSK9Δ31-152(S386A)-FLAG or pCMV-PCSK9Δ31-152(D374Y-S386A)-FLAG as above. Colonies surviving neomycin/hygromycin selection were assessed for PCSK9 secretion by immunoblot analysis of the medium with anti-FLAG M2 antibody. FLAG-tagged human PCSK9, PCSK9(D374), *trans*-PCSK9, *trans*-PCSK9(S386A),

and *trans*-PCSK9(D374Y-S386A) were purified from stably expressing HEK 293S cells as described (3).

Antibodies and Immunoblot Analysis─Mouse monoclonal antibodies were generated against purified recombinant human PCSK9 proteins as described (4). Additional monoclonal antibodies used include: anti-PCSK9 IgG-15A6 (3); anti-human LDLR IgG-HL1 (5); anti-human transferrin receptor (Zymed Laboratories); anti-V5 (Invitrogen); anti-FLAG M2 (Sigma-Aldrich); anti-Actin AC40 (Sigma-Aldrich). The secondary antibody used for immunoblot analysis was horseradish peroxidase-conjugated donkey anti-mouse IgG (Jackson ImmunoResearch Laboratories) with detection using SuperSignal West Pico Chemiluminescent Substrate System (Pierce). LDLR protein was quantified after scanning the film using ImageJ software (Wayne Rasband, Research Services Branch, National Institute of Mental Health, Bethesda, Maryland, USA) to calculate the mean pixel value of LDLR and transferrin signal. Transferrin was used as an invariant protein to correct for differences in sample loading.

Ligand blotting─Ligand blotting of purified human LDLR was performed as described with the buffer described in supplemental data (3) except all steps were performed in buffer containing 50 mM Tris-maleate at pH 6.0, 90 mM NaCl, 2 mM $CaCl_2$, 2.5% (w/v) milk, and the monoclonal anti-FLAG antibody M2 (2.5 μg/ml) was used in place of IgG-15A6 to detect PCSK9.

REFERENCES

1. Sambrook, J., and Russell, D. W. (2001) *Molecular cloning: a laboratory manual*, 3rd Ed. 3 vols., Cold Spring Harbor Laboratory Press, New York
2. Park, S. W., Moon, Y.-A., and Horton, J. D. (2004) *J. Biol. Chem.* **279**, 50630-50638
3. Lagace, T. A., Curtis, D. E., Garuti, R., McNutt, M. C., Park, S. W., Prather, H. B., Anderson, N. N., Ho, Y. K., Hammer, R. E., and Horton, J. D. (2006) *J. Clin. Invest.* **116** 2995-3005.
4. Zhao, Z., Tuakli-Wosornu, Y., Lagace, T. A., Kinch, L., Grishin, N. V., Horton, J. D., Cohen, J. C., and Hobbs, H. H. (2006) *Am. J. Hum. Genet.* **79**, 514-523.
5. van Driel, I. R., Goldstein, J. L., Sudhof, T. C., and Brown, M. S. (1987) *J. Biol. Chem.* **262**, 17443-17449
